# Charge reservoir as a design concept for plasmonic antennas

**DOI:** 10.1515/nanoph-2025-0421

**Published:** 2025-12-09

**Authors:** Rostislav Řepa, Michal Horák, Tomáš Šikola, Vlastimil Křápek

**Affiliations:** Brno University of Technology, Brno, Czechia

**Keywords:** plasmonics, plasmonic antenna, electric near field

## Abstract

Plasmonic antennas exploit localized surface plasmons to shape, confine, and enhance electromagnetic fields with subwavelength resolution. The field enhancement is contributed to by various effects, such as the inherent surface localization of plasmons or the plasmonic lightning-rod effect. Inspired by nanofocusing observed for propagating plasmons, we test the hypothesis that plasmonic antennas with a large cross-section represent a large charge reservoir, enabling large induced charge and field enhancement. Our study reveals that a large charge reservoir is accompanied by large radiative losses, which are the dominant factor, resulting in a low field enhancement.

## 1 Introduction

Plasmonic antennas [1] (PAs) are metallic nanostructures allowing for subwavelength control of light. They are particularly attractive for their ability to locally reshape the driving field (often a plane electromagnetic wave), including its significant confinement and enhancement. The locally enhanced field is exploited in numerous applications, including plasmon-enhanced spectroscopy [2], [3], [4], [5], [6], energy harvesting [7], strong light–matter coupling [8], [9], or sensing [10], [11], [12]. The mechanism of the field enhancement consists in the excitation of localized surface plasmons (LSPs), quasiparticles formed by oscillations of the free electron gas in a metal and the induced electromagnetic field.

The design of PAs with desired properties and their optimization for specific applications exploit various concepts, including plasmonic circuit models [13], [14], [15], [16], transformation optics [17], [18], hybridization models [19], [20], or Babinet’s principle [21], [22], [23], [24]. A particularly inspiring concept is nanofocusing [25], [26], [27], [28], [29], [30], [31], [32], originally formulated for *propagating* surface plasmon polaritons: When they propagate in the plasmonic structure with a decreasing cross-section (e.g., a tapered waveguide or a metallic tip for near-field microscopy), the electromagnetic energy is concentrated. The role of the nanofocusing for LSPR supported by PAs is less clear. The effect of the narrow part is well known. The so-called plasmonic lightning-rod effect [33], [34], [35], [36], [37], [38] states that the induced electric field is particularly strong at the sharp parts of PAs (differences between the electrostatic and plasmonic lightning-rod effects are discussed in Ref. [38]). However, the broad part of the PA is only rarely considered, although it is an essential part of nanofocusing. Several exceptions are represented by a comparison of a bowtie PA and a nanorod dimer PA, showing a stronger field enhancement of the latter [39], a study focusing on the effect of a vortex angle in a triangular PA [40], a comparison of the split ring resonator and crescent resonator showing a stronger field of the latter [41], and a comparison of the field enhancement achieved through nanofocusing and with a PA [42]. However, a systematic study of nanofocusing in PAs, including the analysis of the broad part, is missing.

We propose a hypothesis that the broad part can act as a reservoir of the charge for plasmonic oscillations; when driven by an external excitation, the broad part provides electrons that can be concentrated at the narrow part, forming a large induced charge accompanied by a large electric near field. In our paper, this hypothesis is tested and invalidated.

## 2 Charge reservoir

The central question addressed in our work reads: Can we expect a stronger response of plasmonic antennas if they contain more electrons, under otherwise identical circumstances? In this section, we will explain the motivation for this question, refine it, and design a study to find the answer.

**Figure 1:**
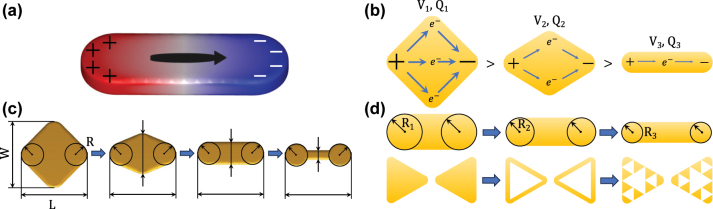
Charge reservoir effect. (a) A scheme of a dipole LSPR supported by a plasmonic nanorod. The current is represented by the black arrow, thickened in the central part of the PA to mark the antinode of the current wave. The antinodes of the induced charge at the peripheral parts of the PA are labeled by + and − symbols. (b) Conceptual explanation of the charge reservoir. From left to right, the cross-section of PAs decreases, resulting in expectedly lower magnitudes of induced charge (represented by decreased size of + and − symbols). (c) The design of the study. From left to right, a diamond PA is transformed through a tapered-diamond PA into a nanorod PA and then to a dumbbell PA by a sequential thinning of the central part, accompanied by a reduction in the charge reservoir. The black circles at the peripheral part of all PAs demonstrate that the radius of curvature is preserved for all PAs. (d) Two sets of PAs not suitable for studying the charge reservoir effect. Top: The nanorods of different widths also differ in the radius of curvature. Bottom: Three bowtie PAs (full, contour, and fractal) differ in the charge oscillation patterns.

We will consider only planar PAs that can be fabricated by the lithography of a thin metallic film. The amount of electrons within the PA will be controlled only by its shape (i.e., the geometrical cross-section); alteration of the PA material is not considered. We will focus on a dipole LSPR, which can be visualized as an oscillating electric dipole, with the antinodes of the oscillating charge at peripheral parts of the PA and the current antinode in the central part, as schematically shown in Figure 1(a).

Figure 1(b) explains the idea of the charge reservoir. Three PAs differing in the cross-section and identical otherwise are subject to the same excitation (a plane electromagnetic wave with the polarization parallel to the excited dipole). In the PAs with a larger cross-section, the driving field shall affect a large charge, and it is plausible to assume that the charge induced at the peripheral parts would be larger in magnitude, as schematically indicated in Figure 1(b). In other words, the volume of the PA shall act as a *reservoir* of the charge that can contribute to LSPR. We will investigate whether this effect, labeled as the *charge reservoir effect*, really exists, is observable, and can be exploited in specific applications of PAs. To this end, we will carry out a comparative study of PAs with different charge reservoirs.

Multiple effects affect the strength of the response of PAs. To isolate the role of the charge reservoir, it is essential to compare the PAs differing in the charge reservoir under otherwise identical conditions. This includes identical material composition and excitation field. We will also aim to keep the LSPR energy constant, either approximately by fixing the length of the PA, or exactly by adjusting the length to the specific energy. The induced field is enhanced near the curved features of the PAs (so-called lightning-rod effect) [33], [38]. We will thus keep constant the geometrical curvature of those parts of PAs where the field is evaluated. Finally, we will require that all PAs support the lowest-energy LSPR of the same plain-dipole topology. Thus, concepts like contour [43], [44] or fractal [45] PAs with the inner part of the PA removed will not be considered in our study. Although they provide a clear way to modify the charge reservoir, the overall performance would be influenced by additional effects, among which the coupling might be particularly important [44].

In our study, we will take a simple plasmonic rod with rounded tips as the central shape. Then, we will either widen the central part by gradually morphing the rod to a diamond or narrow the central part by morphing the rod to a dumbbell. This set of PAs, schematically represented in Figure 1(c), will be used to investigate the charge reservoir. Figure 1(d) then shows two sets of PAs which exhibit a variation of the charge reservoir combined with a variation of additional factors, which are unsuitable for isolating the role of the charge reservoir.

Finally, we would like to point out that the charge reservoir is primarily an intuitive concept for the design of PAs. In our study, we treat it as an ordinal quality: the individual antennas included in the study can be ordered according to the size of their charge reservoir. The charge reservoir can be in principle related to quantitative metrics such as the volume of the PA or the total charge of free electrons in the PA (equal to the volume of the PA multiplied by the electron density and elementary charge). However, we do not aim to describe the reservoir quantitatively.

## 3 Methods

Our methodology combines experimental electron energy loss spectroscopy (EELS) with electromagnetic simulations. EELS provides qualitative insight into the near field of PAs and verifies the simulations, which provide additional quantities such as a full quantitative characterization of the electric field.

### 3.1 Fabrication of plasmonic antennas

PAs were fabricated by the focused ion beam (FIB) lithography [46]. A gold layer with a thickness of 30 nm was deposited by ion beam sputtering using an optimized deposition rate of 0.1 nm/s [47] onto the standard 30-nm-thick silicon nitride membranes for transmission electron microscopy (Agar Scientific). FIB milling was performed using the dual-beam FIB/SEM microscope Tescan LYRA3 with the gallium ion beam energy set to 30 keV and the beam current set to 1 pA (lowest available). PAs were situated in the centre of a metal-free square with the dimensions of 2 × 2 μm^2^, which is sufficient to prevent their interactions with the surrounding metallic frame and the neighbouring PAs.

### 3.2 Electron energy loss spectroscopy

EELS measurements were performed using a FEI Titan S/TEM equipped with a GIF quantum spectrometer. The microscope was operated in scanning monochromated mode at 120 keV, with convergence and collection semi-angles set to 10 and 11.4 mrad, respectively, and spectrometer dispersion set to 0.01 eV/pixel. The probe current was adjusted to around 100 pA, and the full width at half maximum (FWHM) of the zero-loss peak was found to be in the range of 0.10–0.12 eV. To use the full intensity range of the CCD camera in the spectrometer and avoid overexposure, the acquisition time of every spectrum was set to 0.5 ms. Before performing the EELS measurements, the sample was plasma-cleaned in argon–oxygen plasma for 20 s to minimize carbon contamination.

For each PA, we recorded a spectrum image with the spatial dimensions set to 500 × 500 nm^2^ and the pixel size of 2 nm. The raw spectra were averaged over a certain region of interest to reduce the noise. Next, the spectra were normalized to the electron counts of the zero-loss peak (ZLP) (integrated over the energy window from −1 eV to +1 eV) to obtain a quantity proportional to the loss probability per channel, which was further divided by the energy interval of the channel (0.01 eV) to obtain the usual loss probability density (in units 1/eV) (the so-called loss function). Finally, the experimentally determined background and the ZLP (measured at the bare membrane) were subtracted to isolate the contribution of LSPR. For experimental loss function maps, we first integrated the spectrum image over the energy range of 0.1 eV around the resonance energy of an LSPR to obtain a signal map (with reduced noise), which was further divided pixel-wise by the map of the integral intensity of the zero-loss peak. In this case, the zero-loss peak and background were not subtracted.

### 3.3 Electromagnetic simulations

The electromagnetic simulations were performed using the MNPBEM toolbox [48], [49], [50] for Matlab based on the boundary element method (BEM) [51]. The dielectric function of gold was taken from [52]. In all simulations, the membrane was neglected, and the refractive index of the surrounding medium was set to 1. Further details, including the dimensions of all simulated PAs, are provided in Supporting Information, Section S1. The effect of the neglected membrane is discussed in Supporting Information, Section S2.

## 4 Results

In this section, we first validate the simulations by comparing their predictions to experimental data. We then analyze the charge reservoir effect, demonstrating that PAs with a larger charge reservoir generally exhibit a weaker response due to their increased radiative losses. Finally, we discuss the relation between the charge reservoir and specific response functions for various parameters of PAs.

### 4.1 Experimental validation of simulations

**Figure 2:**
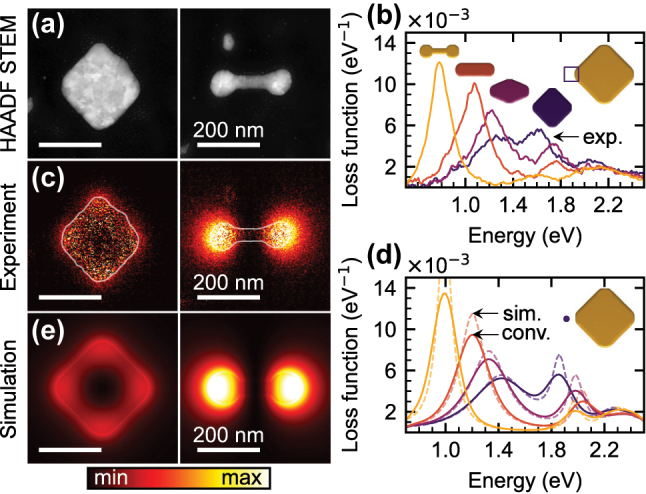
EELS of a set of PAs with a length *L* = 300 nm, a radius *R* = 50 nm, and widths *W* between 300 nm and 40 nm. (a) HAADF STEM image of two PAs with the widths *W* = 300 nm and 40 nm. (b) Experimental loss function spectra for PAs with widths *W* of 40 nm (golden line, dumbbell shape), 100 nm (salmon line, rod shape), 183 nm (maroon line, tapered diamond shape), and 300 nm (indigo line, symmetric diamond shape). The insets above the spectra also indicate the correspondence between the spectra and the shapes. The spectra were averaged over the electron beam positions corresponding to a square next to the left edge of the PA, as schematically indicated in the right inset. (c) Experimental loss function maps recorded for PAs from panel a for the loss energy corresponding to the dipole LSPR (represented by a peak in the corresponding spectra in panel b between 1.0 and 1.4 eV). (d) Theoretical loss function spectra analogous to panel b. The electron beam position, 20 nm from the left edge of PAs, is indicated in the right inset. The dashed lines represent raw simulated spectra, the solid lines show the spectra convolved with a Gaussian with a full width at half maximum (FWHM) of 0.15 eV representing the instrumental broadening of our EELS setup. (e) Theoretical loss function maps analogous to panel c.

We focused on a set of planar PAs [diamonds, a rod, dumbbells as shown in Figure 1(c)] with the identical length *L* = 300 nm, radius *R* = 50 nm, and thickness of 30 nm, while the width *W* of PAs was varied from 300 nm to 40 nm, with *W* > 100 nm corresponding to the (tapered) diamond shape, *W* = 100 nm to the rod shape, and *W* < 100 nm to the dumbbell shape. The PAs were fabricated and analyzed experimentally by EELS. The EELS was also theoretically modelled. The results of this analysis are summarized in Figure 2.

Figure 2(a) shows a high-angle annular dark-field (HAADF) STEM image of two PAs of the set with *W* = 300 nm (a symmetric diamond) and *W* = 40 nm (a dumbbell). The experimental loss function is displayed as a spectrum for a specific position of the electron beam in Figure 2(b) and as a map for a specific value of the energy loss (corresponding to the dipole LSPR) in Figure 2(c). The loss function spectra in Figure 2(b) are shown for a symmetric diamond (*W* = 300 nm), a tapered diamond (*W* = 183 nm), a rod (*W* = 100 nm), and a dumbbell (*W* = 40 nm), as indicated by insets above each spectrum. To suppress experimental noise, the spectra were averaged over a range of electron beam positions spanning a square next to the left edge of the PA, as schematically indicated in the right inset of Figure 2(b). In the following, we focus on the lowest-energy peak of the spectra (with the energy ranging from 1.0 eV to 1.4 eV). The maps of the loss probability at the energy of this peak are shown in Figure 2(c), and assign this peak to a dipole LSPR [20]. The calculated spectra and maps shown in Figure 2(d) and (e) closely match the experimental data, verifying the correctness and accuracy of the simulations and allowing us to use the simulations to retrieve quantities inaccessible experimentally, in particular, the induced electric field. We have convolved the calculated EEL spectra with a Gaussian (FWHM of 0.15 eV) to account for the instrumental broadening. We note that the dimensions of the fabricated PAs are slightly smaller (by about 10 %) than the nominal values considered in simulations.

The second peak in the loss function spectra (Figure 2(b) and (d)) with the energy ranging from 1.7 eV to 2.1 eV can be assigned to a longitudinal quadrupole LSPR [20], [53]. This LSPR is dark. It does not couple to the plane-wave excitation, nor does it decay radiatively. It can be excited by the electron beam, but in simulations, it is well spectrally separated from the dipole mode. Therefore, it will not be considered in further analysis.

### 4.2 Radiative losses and total plasmon charge

**Figure 3:**
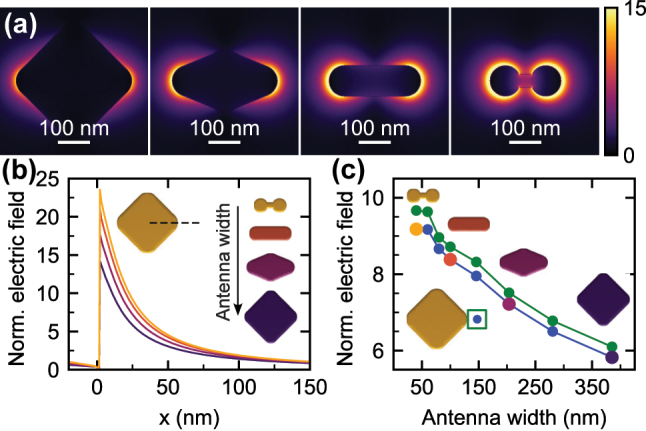
Calculated magnitude of the induced electric field of PAs illuminated with a plane wave at the energy of the dipole LSPR (1.2 eV). (a) A planar cross-section in the middle of the PA height for PAs with the widths of 385 nm (indigo line, symmetric diamond shape), 204 nm (maroon line, tapered diamond shape), 100 nm (salmon line, rod shape), and 40 nm (golden line, dumbbell shape). (b) A linear cross-section along the long axis of PAs (indicated in the inset) for the same PAs as in panel a. The distance from the PA edge is labeled *x*. (c) The magnitude of the field at a distance of 25 nm from the PA edge (blue) or averaged over a cube with a side of 50 nm centered at a distance of 25 nm from the PA edge (green). The fields are normalized to the amplitude of the incident plane wave. The inset shows the position/area where the field is evaluated. The symbols in panel c corresponding to the PAs included in panel b are enlarged and displayed using corresponding colors.

**Figure 4:**
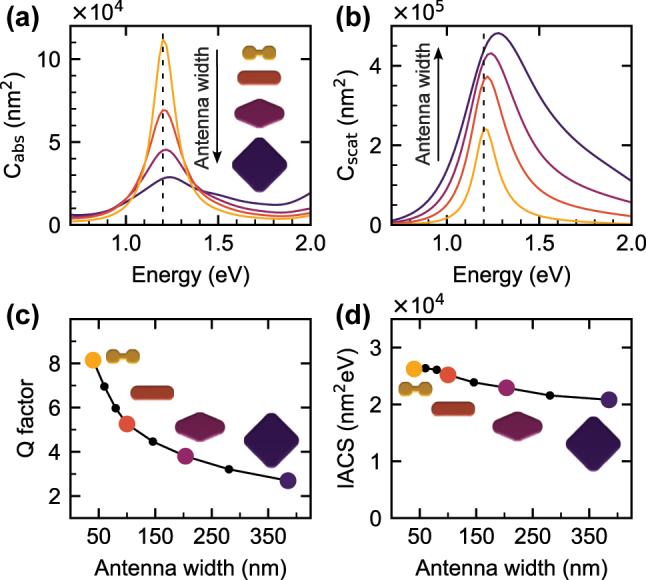
Calculated optical response of a set of PAs with a length adjusted for the energy of the dipole LSPR of 1.2 eV, a radius *R* = 50 nm, and widths *W* between 385 nm and 40 nm. (a) Absorption cross-section for PAs with widths *W* of 40 nm (golden line, dumbbell shape), 100 nm (salmon line, rod shape), 204 nm (maroon line, tapered diamond shape), and 385 nm (indigo line, symmetric diamond shape). The insets on the right side of the spectra also indicate the correspondence between the spectra and the shapes. (b) Scattering cross-section for the same set of PAs. (c) Q factor. (d) Integral absorption cross-section (IACS). The symbols in panels c and d corresponding to the PAs included in panels a and b are enlarged and displayed using corresponding colors.

The results of the previous section, displayed in Figure 2, already show that the PAs with a lower charge reservoir exhibit, rather counterintuitively, a stronger response, as can be observed, e.g., by comparing an intense loss function of the dumbbell to a weak loss function of the diamond in Figure 2(c). However, there are two factors, besides the charge reservoir, that might contribute to this observation. (i) The energy of the dipole LSPR differs among the PAs involved in the comparison. (ii) The electron beam is a localized excitation, with a characteristic decay length [54] of 120 nm for the energy transfer of 1.2 eV and the electron energy of 120 keV utilized in our study. To exclude the effect of these factors, we have adjusted the length of the PAs so that they all exhibit the identical energy of the dipole LSPR of 1.2 eV, and we utilized excitation with a plane electromagnetic wave polarized along the long axis of PAs. The results are summarized in Figures 3 and 4.

The induced electric field is shown in Figure 3. Planar maps in Figure 3(a) qualitatively confirm stronger fields in narrower PAs with lower charge reservoirs. This observation is further supported by linear cross-sections shown in Figure 3(b), which demonstrate that the effect affects a whole area of the enhanced near field rather homogeneously. To quantify the effect, we plot the field at a distance of 25 nm from the PA edge and the field averages over a cube with a side of 50 nm centered around the previous spot in Figure 3(c) as functions of width.

Figure 4(a) shows the calculated spectral dependence of the absorption cross-section, which features a single peak centered at the energy of 1.2 eV, corresponding to the dipole LSPR. A behaviour similar to EELS is observed: as the width of a PA increases (and so does the charge reservoir), the amplitude of the absorption peak is reduced and the peak is broadened. This fact is explained by inspecting the scattering cross-section of the PAs (Figure 4(b)), which is markedly larger for wider PAs (with a larger charge reservoir), with a consequence of large radiative losses, which act as an additional damping channel for LSPR, decreasing and broadening the response function. This is further corroborated by the Q factor (Figure 4(c)) obtained as a ratio of the central energy and the FWHM of the absorption peak (obtained by fitting a Lorentzian to the absorption cross-section), which is pronouncedly reduced for wider PAs. Similarly, enhanced radiative losses explain the reduced field in PAs with a higher charge reservoir, as observed in Figure 3. We note that the positive correlation between the size of a PA and its scattering is well known [39], [40].

Optical response functions of PAs exhibit characteristic resonant spectral dependences. Increased radiative losses result in a decrease and broadening of resonance curves. It might be instructive to complement the analysis of the peak values with the analysis of spectral integrals of the response functions over a full range of the dipole LSPR. As an illustration, we will consider a crude model of an LSPR as a damped harmonic oscillator with a charge *q*, a mass *m*, a resonance frequency *ω*_0_, and a Q factor *Q*, driven by a harmonic field *E*(*t*) = *E*_0_ exp(−*iωt*). The power *P*(*ω*) absorbed by the oscillator reads

(1) $$P\left(\omega \right)=\frac{{\left(qE_{0}\right)}^{2}}{2m\omega_{0}}Q\frac{\gamma^{2}\omega^{2}}{{\left(\omega_{0}^{2}-\omega^{2}\right)}^{2}+\gamma^{2}\omega^{2}},$$

where *γ* = *ω*_0_/*Q*. The peak absorbed power is proportional to the Q factor, *P*_max_ ∼ *Q*, and the FWHM is equal to *γ* and is thus inversely proportional to the Q factor. The spectral integral of the power, ∫*P*(*ω*)d*ω*, is in this crude model approximately independent of the Q factor, and thus independent of the charge reservoir. However, in more realistic models, or for different response functions, the spectral integral might depend on the charge reservoir, providing additional insight into the relation between the charge reservoir and the response of PAs. In the following, we will classify the optical response functions into three categories: positively correlated to the charge reservoir if both the peak value and the spectral integral increase with increasing charge reservoir, negatively correlated to the charge reservoir if both the peak value and the spectral integral decrease with increasing charge reservoir, and partially correlated to the charge reservoir if the peak value decreases and spectral integral increases with increasing charge reservoir. We exploit this approach in Figure 4(d), where we inspect the spectral integral of the absorption cross-section. Remarkably, the spectral integral is nearly identical for all involved PAs, just as predicted by the crude oscillator model.

**Figure 5:**
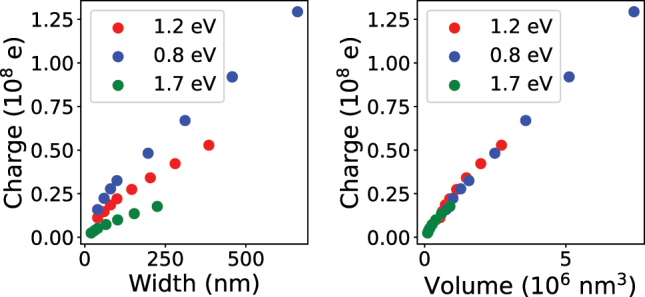
Total charge (in units of 10^8^ elementary charges) involved in plasmon oscillations according to the generalized harmonic oscillator model [55] as a function of the PA width (left) and the PA volume (right) for sets of PAs with the dipole LSPR energy fixed at 1.2 eV (red symbols), 0.8 eV (blue symbols), and 1.7 eV (green symbols).

Kats *et al.* [55] proposed a generalized harmonic oscillator model that approximately accounts for the radiative losses and the total charge involved in the plasmonic oscillations. We have applied the model to the calculated scattering and absorption cross-sections from Figure 4 and to two other sets of PAs with the dipole LSPR energy fixed at 0.8 eV and 1.7 eV. The parameters of the model were determined by fitting both cross-sections simultaneously, yielding a very good agreement between the data and model. The details of the model and the fitting procedure are discussed in Supporting Information, Section S3. The total charge involved in plasmonic oscillations shown in Figure 5 correlates with the width or the volume of PAs. Particularly prominent is the dependence on the volume, which is universal for all three sets of the PAs. In this way, the existence of the expected charge reservoir is demonstrated: The charge of the free electron participating in plasmon oscillations indeed scales with the volume of PAs. However, it is important to stress the limits of both the damped harmonic oscillator and the generalized oscillator models. Both models neglect significant aspects of LSPs, including the spatial distributions of induced charge, current, and the electromagnetic field. We therefore base our analysis entirely on the electrodynamic simulation results and use the models only to facilitate the interpretation of those simulations.

We will now investigate in more detail the relation between the charge reservoir and optical response functions.

### 4.3 Optical response functions

**Figure 6:**
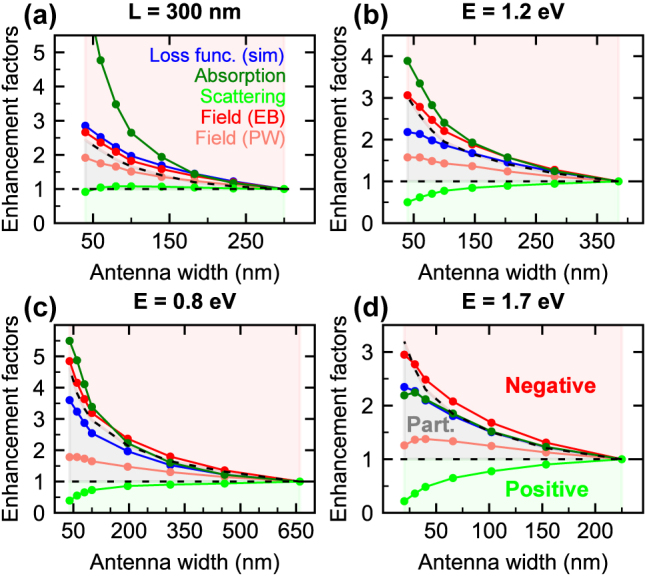
Enhancement factors of selected Figures of Merit (FoMs) defined as the ratio of the values for a specific PA and for a reference PA, which we set as a symmetric diamond: Theoretical peak loss probability (dark blue), peak absorption cross-section (dark green), peak scattering cross-section (light green), magnitude of the electric field at a distance of 25 nm from the edge of PA for the electron beam excitation (dark red) and the plane wave excitation (light red). Four sets of PAs with different widths are compared. (a) A set of PAs with the length fixed at 300 nm. (b–d) Sets of PAs with the dipole LSPR energy fixed at (b) 1.2 eV, (c) 0.8 eV, and (d) 1.7 eV. The dashed black lines correspond to the enhancement factor of 1 and the enhancement factor of the Q factor, and demarcate the areas of a positive correlation with the charge reservoir (light-green shaded area), a partial correlation with the charge reservoir (light-blue shaded area), and a negative correlation with the charge reservoir (light-red shaded area). For clarity, the three areas are labeled in panel (d) as positive (positive correlation with the charge reservoir), part (partial correlation with the charge reservoir), and negative (negative correlation with the charge reservoir).

The performance of PAs in various applications is characterized by the so-called figures of merit (FoMs). Here we address the correlation between the charge reservoir and prominent FoMs: peak loss probability (i.e., the maximum value observed at the energy of the dipole LSPR), peak absorption and scattering cross-sections, and magnitude of the field at a specific distance from the PA edge (set to 25 nm) for both electron beam and plane wave excitations. All FoMs are retrieved from simulations. To compare the role of the charge reservoir for different FoMs, we represent them by dimensionless enhancement factors, defined as a ratio of the FoM’s values for a specific PA and a reference PA (we use the symmetric diamond as the reference). The values of the enhancement factors are shown in Figure 6 for four sets of PAs: with a fixed length *L* = 300 nm (Figure 6(a)) and with a fixed energy of the dipole LSPR of 1.2 eV (Figure 6(b)), 0.8 eV (Figure 6(c)), and 1.7 eV (Figure 6(d)).

The analyzed FoMs can be classified into three categories: positively correlated to the charge reservoir if both the peak value and the spectral integral increase with increasing charge reservoir, negatively correlated to the charge reservoir if both the peak value and the spectral integral decrease with increasing charge reservoir, and partially correlated to the charge reservoir if the peak value decreases and spectral integral increases with increasing charge reservoir. Those positively correlated with the charge reservoir exhibit an enhancement factor lower than 1 (the reference value for the symmetric diamond is higher than the actual value for the other PA). There is only one such FoM: the scattering cross-section. Since it is demanding to calculate the spectral dependences of the induced field, we will introduce an approximate procedure to distinguish between the partial and negative correlation, assuming that the spectral integral is proportional to the peak value divided by the Q factor. Consequently, the FoMs with the enhancement factor smaller/larger than the enhancement factor of the Q factor will be classified as partially/negatively correlated to the charge reservoir, respectively. Remarkably, the induced electric field for the plane wave excitation is partially correlated to the charge reservoir. We speculate that if the radiative losses are suppressed, e.g., by employing high-Q plasmon resonances [56], the positive correlation between the induced electric field and the charge reservoir might be enabled.

It is noteworthy that previous findings are rather general. We include in our analysis two additional sets of PAs: small ones with suppressed radiative losses (the energy of the dipole LSPR was fixed at 1.7 eV, corresponding lengths varied between 110 nm and 225 nm), and large ones which are closer to a perfect conductor regime (the energy of the dipole LSPR was fixed at 0.8 eV, the corresponding dielectric function of gold *ɛ* = −115 + 11*i*). While there are some variations in the values of FoMs, no systematic difference was observed.

Next, we discuss our results in the context of recent progress in the field. Numerous studies consistently report a positive correlation between radiation losses and the nanostructure volume [57], [58], [59], [60], in agreement with our findings. However, no study so far has addressed a direct comparison of optical response at a fixed resonance energy and fixed curvature of the antenna terminations. This distinction is crucial. In our work, we systematically vary the charge reservoir (i.e., the antenna volume) while keeping both resonance energy and end curvature constant. This enables us to isolate the role of the charge reservoir size in determining both radiative losses and field enhancement.

Finally, we would like to discuss the similarities and differences between the nanofocusing of propagating plasmons in tapered plasmonic waveguides (or similar structures) and the charge reservoir effect in PAs. Both cases involve tapered geometries. The taper angles are typically, but not necessarily, smaller in the case of nanofocusing. It has been demonstrated that adiabatic nanofocusing occurs in gold up to the taper angle of 35°, and that non-adiabatic nanofocusing occurring at larger taper angles still yields a large field enhancement [61]. A significant difference is presented by the length of the plasmonic structure. The optimum taper length for nanofocusing of around 10 μm has been found for gold at 633 nm (1.96 eV) [61], while the length of PAs operating at the same energy can be estimated as 100–200 nm, depending on the precise geometry (our PAs operating at 1.7 eV have the lengths between 110 nm and 225 nm). Consequently, the tapers used for nanofocusing support a quasicontinuous spectrum of plasmons, which results in their broadband functionality [62], [63], [64]. On the other hand, PAs support a discrete spectrum of resonances. Another significant difference is constituted by the radiative losses, which are negligible in the case of nanofocusing but play a decisive role in the case of the charge reservoir effect, as we have demonstrated in this study.

## 5 Conclusions

We have tested the hypothesis that the response of the plasmonic antennas can be enhanced by their large charge reservoir. The charge reservoir was related to the volume of the plasmonic antenna containing the free electron gas collectively responding to the driving electromagnetic field. The large charge reservoir (typically achieved through a large cross-section of the plasmonic antenna) might then, under otherwise identical conditions, provide large induced charges, fields, and the optical response functions such as scattering and absorption cross-sections. Our analysis, which is based on a comparative study of plasmonic antennas with varied charge reservoirs but fixed curvatures and plasmon resonance energies, revealed that only the scattering cross-section is positively correlated to the charge reservoir. This, in turn, enhances the radiative losses which result in the negative correlation between the charge reservoir and the peak values of the induced fields and response functions. On the other hand, spectral integrals of the induced field are positively correlated to the charge reservoir.

The charge reservoir effect is analogous to the nanofocusing observed in propagating surface plasmon polaritons. The principal difference stems from the non-radiative character of propagating plasmon polaritons, which opens the possibility for the field enhancement through nanofocusing.

We conclude that the charge reservoir effect can be exploited as the design principle for plasmonic antennas with an efficient coupling to radiative modes, but, somewhat counterintuitively, not for the large field enhancement.

## Supplementary Material

Supplementary Material Details
