# Supplementary material for: Charge reservoir as a design concept for plasmonic antennas
Source: Nanophotonics. 2025 Dec 9;14(25):4637–45. doi: 10.1515/nanoph-2025-0421 (PMC12714058; doi:10.1515/nanoph-2025-0421)
Supplement: Supplementary file 1 — Supplementary Material Details [file j_nanoph-2025-0421_suppl_001.pdf]

Rostislav Řepa, Michal Horák, Tomáš Šikola, and Vlastimil Křápek*

Charge reservoir as a design concept for plasmonic antennas: Supporting Information

S1 Electromagnetic simulations

The electromagnetic simulations were performed using the MNPBEM toolbox [1–3] for MATLAB. The MNPBEM toolbox is based on the boundary element method (BEM), which was adapted to describe electron energy loss spectroscopy (EELS) by F. J. García de Abajo in collaboration with A. Howie [4]. In MNPBEM, Maxwell's equations are solved in the form of surface integral equations at the interfaces between different media. These interfaces are discretized into small boundary elements, assuming that each medium is homogeneous, isotropic, and separated by sharp (abrupt) interfaces. Once the excitation scheme is specified, the BEM equations are solved for the given excitation by computing the auxiliary surface charges and currents. From these surface charges and currents, one can then calculate derived quantities such as absorption and scattering cross sections for the plane-wave excitation, the loss function spectra in EELS simulations, the induced electromagnetic fields, etc.

In our study, we consider planar gold plasmonic antennas (PAs), which are modeled in the toolbox using the *comparticle* function. Two examples of these particle models with discretized boundaries are shown in Fig. S1(a,b). The dielectric function of gold was taken from Ref. [5], and the refractive index of the surrounding medium was set to 1. In all simulations, the BEM solver was configured to include retardation effects, i.e., to solve the full Maxwell equations, since our PAs do not satisfy the conditions required for applying the quasistatic approximation. Two types of incident excitation fields were used: an electron beam and a plane electromagnetic wave.

For the electron beam (EELS) simulations, the beam parameters were identical to those used in the experimental setup. Specifically, the incident beam energy was set to 120 keV, and its position was 20 nm from the left edge of each individual PA. The loss function spectra were calculated by sweeping the energy loss of the incident electron beam. The peaks in these spectra were fitted with Lorentzian profiles to extract the central energies of the dipole LSPR modes for each PA. The loss function maps were obtained by scanning the electron beam position over a defined area around the PA at the peak energy loss of the dipole LSPR. These calculations were carried out for a set of PAs (diamond, rod, and dumbbell geometries) with identical length $L = 300$ nm, radius $R = 50$ nm, and thickness of 30 nm, but varying widths (listed in Table S1).

The dipole resonance energies of individual PAs can be tuned by modifying their lengths. A resonance energy of 1.2 eV was found to be achievable for all PA types with reasonable length adjustments. To reach this energy, the lengths of the individual PA types were varied from the nominal value of 300 nm to the values listed in the lower part of Table S1. Note that the widths of the diamond PA and the tapered diamond PA were adjusted accordingly to preserve their geometrical aspect ratios, while the widths of the thinner PAs (rod and dumbbell types) remained unchanged. Similarly, we obtained the dimensions of the PA with the resonance energy fixed at 0.8 eV and 1.7 eV. Due to the size restrictions of PAs with the resonance energy of 1.7 eV, we have adjusted their radius R to 20 nm.

Next, the excitation was changed from the electron beam to a plane electromagnetic wave polarized along the horizontal axis of the PAs. Scattering and absorption spectra were calculated from the induced surface charges and currents by varying the energy of the incident plane wave. For the fixed peak energies, the induced electric fields were also computed and quantified.

S2 Effect of the substrate

Throughout the main paper, the substrate is omitted from the simulations. In this section, we discuss its effect on the loss function spectra. In MNPBEM, the silicon nitride (SiN) membrane used in the exper-

Rostislav Řepa, Brno University of Technology, Brno, Czechia, rostislav.repa@vutbr.cz; 0009-0005-2483-3597
Michal Horák, Brno University of Technology, Brno, Czechia, michal.horak2@ceitec.vutbr.cz; 0000-0001-6503-8294
Tomáš Šikola, Brno University of Technology, Brno, Czechia, sikola@fme.vutbr.cz; 0000-0003-4217-2276
***Corresponding author: Vlastimil Křápek,** Brno University of Technology, Brno, Czechia, krapek@vutbr.cz; 0000-0002-4047-8653

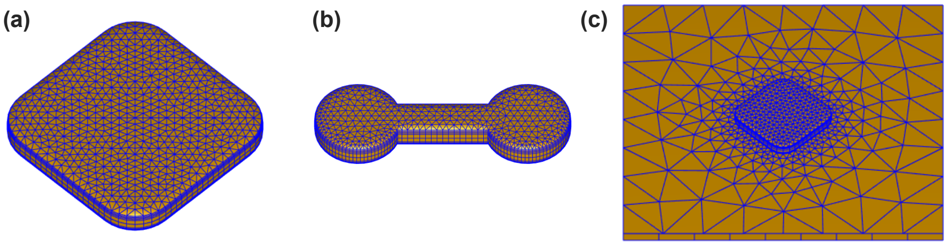

Fig. S1: Examples of plasmonic antenna (PA) geometries used in the simulations, showing their discretized boundaries: (a) diamond PA, (b) dumbbell PA, and (c) diamond PA positioned on a 30-nm-thick SiN substrate.

Tab. S1: Geometrical parameters of the simulated sets of plasmonic antennas presented in the main paper. The thickness of all PAs is set to 30 nm, the radius R is 50 nm for all PAs except the set with the energy of 1.7 eV, where it is 20 nm. For PAs with identical lengths of 300 nm, we provide the energy of the dipole mode determined by fitting the calculated EEL spectra. For PAs involved in Figs. 2, 3, and 4, we provide a link to the spectrum. The relation between the labels and colors is the following: symmetric diamond (golden), tapered diamond (salmon), rod (maroon), dumbbell (indigo).

**Identical lengths (300 nm)**								
Width (nm)	300	234	183	140	100	80	60	40
Energy (eV)	1.41	1.37	1.32	1.27	1.20	1.15	1.08	0.99
link to Fig. 2	golden	–	salmon	–	maroon	–	–	indigo
**Identical energies (1.2 eV)**								
Width (nm)	385	280	204	146	100	80	60	40
Length (nm)	385	370	350	330	305	283	261	236
link to Figs. 3, 4	golden	–	salmon	–	maroon	–	–	indigo
**Identical energies (0.8 eV)**								
Width (nm)	660	457	311	196	100	80	60	40
Length (nm)	660	635	610	585	525	500	460	400
**Identical energies (1.7 eV)**								
Width (nm)	225	154	102	66	40	30	20	
Length (nm)	225	210	190	170	147	130	110	

iments was modeled as two parallel plates separated by a distance corresponding to the membrane thickness (30 nm), on top of which the particle was placed, as shown in Fig. S1(c). The dielectric environment of the SiN membrane can be approximated by a dielectric constant set equal to 4 in the considered spectral region [6].

For comparison, the loss function spectra were calculated for a diamond-shaped PA with a diagonal length of 300 nm, first without the substrate [Fig. S2(a)] and then with the substrate included [Fig. S2(b)]. The electron beam was positioned 20 nm from the left edge of the antenna (indicated by the blue dot), and its incident energy was set to 120 keV. To account for instrumental broadening, mainly due to the finite width of the zero-loss peak, the calculated spectra were convolved with a Gaussian function of 0.15 eV full width at half maximum (FWHM), shown as solid green lines.

Let us first consider the case without the substrate [Fig. S2(a)]. A comparison of the calculated (blue) and experimental (red) spectra shows the following: (1) The calculated spectrum is blue-shifted by approximately 0.15 eV with respect to the experiment. (2) The first two LSPR peaks are slightly more separated in the calculated spectrum (0.44 eV) than in the experimental one (0.36 eV). The convolved spectrum (green line) reproduces the experimental spectrum reasonably well, except for a slight energy offset, which can be primarily attributed to the omission of the substrate. To facilitate comparison, a constant energy shift of 0.2 eV was applied to the convolved spectrum; the resulting spectrum (green dashed line) closely matches the experimental result.

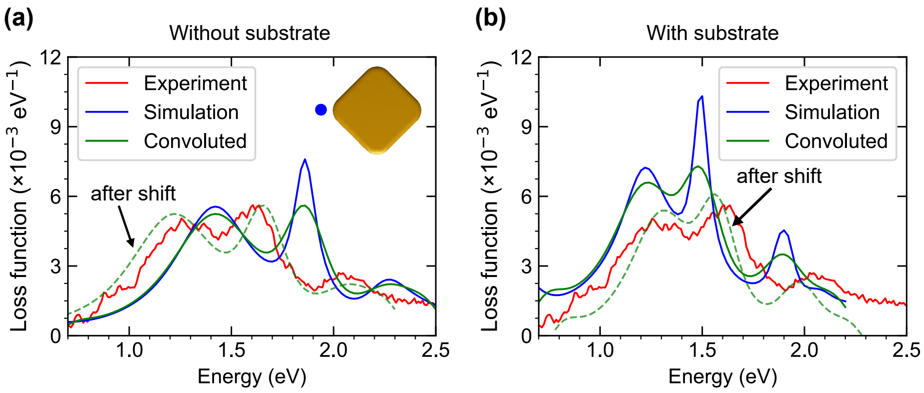

Fig. S2: Comparison of experimental loss function spectra (red lines) with simulated spectra (blue lines) for two models: (a) PA without the substrate and (b) PA on a substrate. The electron beam position is indicated in the inset (blue dot). The calculated spectra were convolved with a Gaussian function (full width at half maximum of 0.15 eV; green line) and manually shifted in energy for comparison (green dashed line).

A similar analysis can be performed for the case with the substrate included [Fig. S2(b)]. The following differences can be observed: (1) The calculated spectrum is red-shifted by approximately 0.1 eV relative to the experiment. This energy shift may arise from by differences between the real and model dimensions of the PA or from variations in the real and model dielectric functions. (2) The peaks in the calculated spectrum are slightly less separated (0.28 eV) than in the experimental one (0.36 eV). (3) The calculated spectrum exhibits a higher background signal, which in the experiment is partially removed by subtracting the membrane contribution. As before, the calculated spectrum was convolved with the Gaussian function (green line) and shifted in both energy (by 0.1 eV) and intensity (by 2×10^{-3} eV^{-1}) to enable direct comparison with the experiment (green dashed line).

In summary, the comparison of experimental and simulated loss function spectra demonstrates that the overall spectral features are well reproduced even when the substrate is omitted from the simulations, with only a minor shift in the peak energies. Including the substrate introduces additional numerical complexity and potential inconsistencies in the calculations, particularly due to the thin dielectric layer and its interface treatment. Therefore, to ensure numerical stability and focus on the plasmonic behavior of the antennas, the substrate was omitted from all simulations presented in the main paper.

S3 Generalized harmonic oscillator model

Kats *et al.* [7] proposed a plasmon model in which a body of electrons with the charge q, the mass m, and the displacement $x(t)$ is driven by the harmonic external electric field $E_0 \exp(i\omega t)$ and experiences the restoring force $m\omega_0^2$, the internal damping $\Gamma_a \mathrm{d}x/\mathrm{d}t$, and the radiative reaction force $\Gamma_s \mathrm{d}^3x/\mathrm{d}t^3$ with $\Gamma_s = q^2/6\pi\varepsilon_0 c^3$. The equation of the motion thus reads

$$m\frac{\mathrm{d}^2x}{\mathrm{d}t^2} + \Gamma_a\frac{\mathrm{d}x}{\mathrm{d}t} + m\omega_0^2 x = qE_0\exp(i\omega t) + \Gamma_s\frac{\mathrm{d}^3x}{\mathrm{d}t^3}. \quad \text{(S1)}$$

The amplitude of the displacement x_0 follows

$$|x_0(\omega)|^2 = \frac{q^2}{m^2}\frac{E_0^2}{(\omega_0^2-\omega^2)^2 + (\omega^2/m^2)(\Gamma_a + \omega^2\Gamma_s)^2}. \quad \text{(S2)}$$

Finally, considering the usual expression for the plane wave intensity

$$I_0 = \frac{E_0^2}{2\mu_0 c}, \quad \text{(S3)}$$

we obtain the absorption cross-section

$$C_{\mathrm{abs}}(\omega) = 2\mu_0 c\omega^2\Gamma_a\frac{|x_0(\omega)|^2}{E_0^2}, \quad \text{(S4)}$$

and the scattering cross-section

$$C_{\mathrm{scat}}(\omega) = 2\mu_0 c\omega^4\Gamma_s\frac{|x_0(\omega)|^2}{E_0^2}. \quad \text{(S5)}$$

Both cross-sections contain four free parameters (ω_0, q, m, Γ_a) that can be determined by fitting the functions (Eqs. S4 and S5) to the simulated data.

We have slightly adapted the model to our purposes. First, we have noticed that the model is overparameterized. The three parameters, q, m, and Γ_a, are included in Eqs. S4 and S5 only in two expressions, q^2/m and Γ_a/m. It is thus impossible to determine all three parameters. Instead, we determine the total mass of the electrons as $m = Nm^*m_0$, where the number of electrons $N = q/e$ (e is the elementary charge), m_0 is the electron mass, and for the effective mass of gold m^* we take the value 1.35 reported in Ref. [7].

Further, we included a phenomenological amplitude factor C_0, which is needed for a reliable reproduction of the amplitude of both cross-sections. The need for this factor can stem primarily from the overly simple oscillator model that neglects, among other factors, the spatial distribution of the induced electromagnetic field. Further need for C_0 might arise from the limited accuracy of the numerical simulations yielding the data to which the model is fitted. The adapted spectral dependences of the optical cross-sections read

$$C_{\text{abs}}(\omega) = 2C_0\mu_0 c\omega^2\Gamma_a \frac{|x_0(\omega)|^2}{E_0^2}, \tag{S6}$$

$$C_{\text{scat}}(\omega) = 2C_0\mu_0 c\omega^4\Gamma_s \frac{|x_0(\omega)|^2}{E_0^2}. \tag{S7}$$

with free parameters C_0, ω_0, q, and Γ_a.

The models of the absorption and scattering cross-sections (Eqs. S6 and S7) were fitted to the simulated cross-sections shown in Fig. 4(a,b) of the main paper and similar data obtained for other sets of PAs. The accuracy of the models is very good, as demonstrated in Fig. S3 for the instance of two PAs with the dipole LSPR energy of 1.2 eV. This figure clearly shows that the optical response in the broad spectral range can be assigned to a single dipole LSPR, confirming our assignment of the higher-order peak in the EELS spectrum [Fig. 2(b,d) in the main paper] to a dark mode. Minor deviations above the energy of 1.8 eV (a decrease in the calculated scattering cross-section and an increase in the calculated absorption cross-section compared to the model quantities) can be attributed to the onset of the interband absorption of gold, which is not included in the oscillator model.

The values of the estimated parameters are listed in Table S2 together with the PA widths (that can be used to link the records in Table S2 to the geometrical parameters in Table S1). The values ω_0 and q are rather realistic. For example, for the PA set with the dipole LSPR energy fixed to 1.2 eV, the values of ω_0 correspond to the realistic energies between 1.21 eV and 1.33 eV. The charge of the diamond PA shown in Fig. S3 equals to 4.8×10^7 elementary charges, while by multiplying the volume of the diamond PA (2.7×10^6) by the carrier density of gold of 5.9×10^{22} cm^{-3} reported by Kats et al. [7] yields total charge of free electrons in the PA of 15.9×10^7 elementary charges, i.e., around 30 % of free charges in the PA contribute to the plasmonic response. Taking into account the approximate model, the existence of the skin effect, or the neglected spatial distribution of the induced electromagnetic field in the model, this represents a reasonable agreement. All parameters except for the charge q are only weakly dependent on the volume of PAs.

S4 Mode analysis

Our analysis, including the FoMs of Fig. 6 and generalized harmonic oscillator model, assumes that the observed response function can be fully attributed to a single dipole LSPR. However, the asymmetrical profiles of the scattering cross-sections – most apparent for the diamond-shaped and tapered antennas in Fig. 4(b) of the main text – could imply the excitation of higher-order modes. In this section, we provide clear evidence that the response is indeed related to the single dipole LSPR.

We inspect the character of the mode by calculating the electric fields at three distinct energies along the spectral profile. We selected the set of PAs tuned to the resonance energy of 1.2 eV, which is the primary focus of the main paper. Figure S4 presents the imaginary part of the z-component of the induced electric field, evaluated in a planar cross-section located 20 nm above the top surface of the PAs. Following Gauss' law, this field is proportional to the induced charge in the PA. The nodal planes in the spatial distribution of the z-component of the induced electric field correspond to the nodal plane in the distribution of the induced charge, allowing us to assess the nature of the mode.

Results are shown for both the diamond-shaped antenna [Figs. S4(a–c)] and the tapered diamond antenna [Figs. S4(d–f)]. In all cases, the field distributions exhibit the characteristic features of a longitudinal dipole mode, with opposite signs of the z-component of the electric field at the two antenna terminations (corresponding to opposite charge signs). As we increase the energy from the resonance energy of 1.2 eV [Fig. S4(a) and Fig. S4(d)], the magnitude of the field decreases, but its spatial distribution remains qualitatively unchanged. Thus, we can conclude that in the

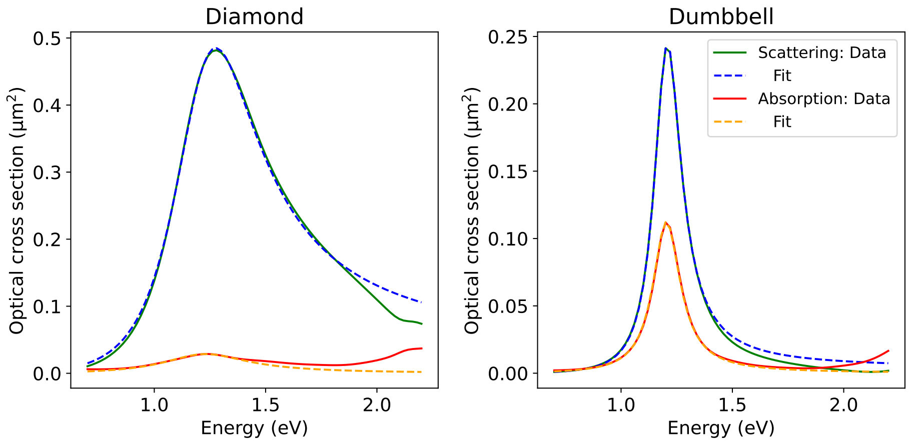

Fig. S3: Calculated scattering cross-section (green solid line) fitted with the model of Eq. S7 (blue dashed line) and calculated absorption cross-section (red solid line) fitted with the model of Eq. S6 (orange dashed line) for the PAs with the dipole LSPR energy of 1.2 eV: (left) A diamond with a width of 385 nm. (right) A dumbbell with a width of 40 nm.

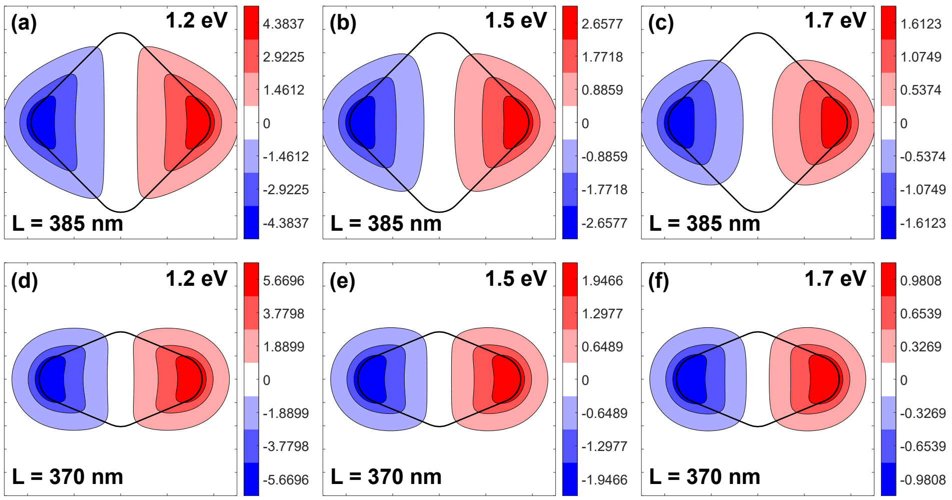

Fig. S4: Imaginary part of the z-component of the induced electric field. Planar cross-section taken 20 nm above the diamond-shaped PA illuminated with a plane wave at (a) 1.2 eV (resonance), (b) 1.5 eV, and (c) 1.7 eV. Corresponding fields for the tapered diamond PA at (d) 1.2 eV (resonance), (e) 1.5 eV, and (f) 1.7 eV.

Tab. S2: Parameter estimators of the generalized harmonic oscillator model.

**Identical energies (1.2 eV)**								
Width (nm)	385	280	204	146	100	80	60	40
Volume (10^6 nm^3)	27.2	19.9	14.8	11.4	8.88	7.34	6.26	5.49
q (10^8 e)	0.47	0.38	0.31	0.25	0.20	0.17	0.13	0.10
$\hbar\omega_0$ (eV)	1.33	1.29	1.26	1.24	1.23	1.22	1.21	1.21
γ_a (10^{13} s^{-1})	4.7	4.9	5.2	5.4	5.8	6.3	6.6	7.3
C_0	0.62	0.58	0.56	0.54	0.53	0.53	0.52	0.52
**Identical energies (0.8 eV)**								
Width (nm)	660	457	311	196	100	80	60	40
Volume (10^6 nm^3)	74.1	51.2	35.8	24.9	15.7	12.8	10.1	7.64
q (10^8 e)	1.16	0.83	0.60	0.43	0.29	0.25	0.20	0.14
$\hbar\omega_0$ (eV)	0.97	0.91	0.87	0.84	0.82	0.81	0.81	0.81
γ_a (10^{13} s^{-1})	4.6	3.9	3.7	3.7	4.4	4.9	5.5	6.3
C_0	0.69	0.61	0.57	0.55	0.54	0.54	0.53	0.52
**Identical energies (1.7 eV)**								
Width (nm)	225	154	102	66	40	30	20	
Volume (10^6 nm^3)	8.99	6.01	3.96	2.68	1.84	1.41	1.09	
q (10^8 e)	0.16	0.12	0.089	0.066	0.045	0.034	0.022	
$\hbar\omega_0$ (eV)	1.74	1.72	1.71	1.70	1.70	1.70	1.70	
$gamma_a$ (10^{13} s^{-1})	7.4	7.5	7.8	8.2	8.7	9.1	9.6	
C_0	0.56	0.55	0.54	0.53	0.52	0.52	0.51	

considered spectral region the plasmonic response of our PAs can be, indeed, attributed to a single mode – the longitudinal dipole. In addition, we would like to stress that the asymmetric shape of the scattering cross-section can be explained by the prefactor ω^4 in its spectral dependence (see Eq. S7).

References

[1] U. Hohenester and A. Trügler, "MNPBEM – a Matlab toolbox for the simulation of plasmonic nanoparticles," Comput. Phys. Commun., vol. 183, no. 2, pp. 370–381, 2012.

[2] U. Hohenester, "Simulating electron energy loss spectroscopy with the MNPBEM toolbox," Computer Physics Communications, vol. 185, no. 3, pp. 1177–1187, 2014.

[3] J. Waxenegger, A. Trügler, and U. Hohenester, "Plasmonics simulations with the MNPBEM toolbox: Consideration of substrates and layer structures," Comput. Phys. Commun., vol. 193, pp. 138–150, 2015.

[4] F. J. García de Abajo and A. Howie, "Retarded field calculation of electron energy loss in inhomogeneous dielectrics," Phys. Rev. B, vol. 65, p. 115418, 2002.

[5] P. B. Johnson and R. W. Christy, "Optical constants of the noble metals," Phys. Rev. B, vol. 6, pp. 4370–4379, 1972.

[6] F. P. Schmidt, H. Ditlbacher, F. Hofer, J. R. Krenn, and U. Hohenester, "Morphing a plasmonic nanodisk into a nanotriangle," Nano Lett., vol. 14, no. 8, pp. 4810–4815, 2014.

[7] M. A. Kats, N. Yu, P. Genevet, Z. Gaburro, and F. Capasso, "Effect of radiation damping on the spectral response of plasmonic components," Opt. Express, vol. 19, no. 22, pp. 21 748–21 753, 2011.
